# Fitness selection in human pluripotent stem cells and interspecies chimeras: Implications for human development and regenerative medicine

**DOI:** 10.1016/j.ydbio.2021.03.025

**Published:** 2021-08

**Authors:** Jun Wu, Ivana Barbaric

**Affiliations:** a Department of Molecular Biology, University of Texas Southwestern Medical Center, Dallas, TX, 75390, USA; b Hamon Center for Regenerative Science and Medicine, University of Texas Southwestern Medical Center, Dallas, TX, 75390, USA; c Centre for Stem Cell Biology, Department of Biomedical Science, The University of Sheffield, Western Bank, Sheffield, S10 2TN, United Kingdom; d Neuroscience Institute, The University of Sheffield, Western Bank, Sheffield, S10 2TN, United Kingdom

**Keywords:** Human pluripotent stem cells, Cell competition, Interspecies chimeras

## Abstract

A small number of pluripotent cells within early embryo gives rise to all cells in the adult body, including germ cells. Hence, any mutations occurring in the pluripotent cell population are at risk of being propagated to their daughter cells and could lead to congenital defects or embryonic lethality and pose a risk of being transmitted to future generations. The observation that genetic errors are relatively common in preimplantation embryos, but their levels reduce as development progresses, suggests the existence of mechanisms for clearance of aberrant, unfit or damaged cells. Although early human embryogenesis is largely experimentally inaccessible, pluripotent stem cell (PSC) lines can be derived either from the inner cell mass (ICM) of a blastocyst or by reprogramming somatic cells into an embryonic stem cell-like state. PSCs retain the ability to differentiate into any cell type *in vitro* and, hence, they represent a unique and powerful tool for studying otherwise intractable stages of human development. The advent of PSCs has also opened up a possibility of developing regenerative medicine therapies, either through PSC differentiation *in vitro* or by creating interspecies chimeras for organ replacement. Here, we discuss the emerging evidence of cell selection in human PSC populations *in vivo* and *in vitro* and we highlight the implications of understanding this phenomenon for human development and regenerative medicine.

## Highlights

• Cell selection might play a role in human embryo development.
• Pluripotent stem cells provide a platform for studying human development.
• Cell competition occurs in human pluripotent stem cell cultures.
• Cell competition is a barrier to interspecies chimerism.

## 1 Introduction

Cell-cell interactions are at the crux of normal tissue development and function. Mediated by membrane receptors and signals, soluble factors or mechanical cues, cell-cell communication orchestrates development of nascent tissues and regulates homeostasis of mature ones. Remarkably, cell-cell interactions are not always cooperative, but rather, they can also be competitive in nature. A fascinating aspect of cellular interaction is cell competition, a quality control mechanism whereby cells utilise soluble signals, mechanical cues or perform direct fitness comparisons in order to weed out less fit, albeit otherwise viable, cells from their populations (reviewed in (Baker, 2020; Bowling et al., 2019)). In this review, we refer to cell fitness as the ability of a cell to survive and proliferate in a particular environment. Fitter cells are referred to cells that, when comparing to other cells (less fit), are better suited to thrive in the same environment.

Viewed through the prism of quality assurance, it is difficult to imagine a more important context of competitive cellular interactions than that of the early embryo. Upon fusion of gametes and a zygote formation, development progresses through a sequence of cleavage divisions, leading to the rapid and exponential growth in cell numbers. Once the embryo reaches the blastocyst stage, at 5–7 days post-fertilisation, a small number of pluripotent epiblast cells are segregated and destined to produce all of the cell types in the human body. It is therefore reasonable to postulate that selection against unfit cells within the pluripotent population is pivotal for ensuring elimination of mis-patterned or genetically aberrant cells that could otherwise instigate developmental defects or pregnancy loss. Indeed, experimental evidence for the role of cell competition in development has been gathered from many model organisms, including mammalian species (reviewed in (Hashimoto and Sasaki, 2020)). Nonetheless, the role of cell competition in development of human embryos remains unexplored, as studying human embryogenesis presents both ethical and technical challenges.

Attempts to study and model early human development have led, first, to the derivation of human embryonic stem cells (ESCs) from human blastocysts (Thomson et al., 1998) and later, to the generation of induced pluripotent stem cells (iPSCs) by reprogramming somatic cells to an embryonic stem cell-like state (Takahashi et al., 2007). These remarkable populations of cells, collectively termed pluripotent stem cells (PSCs), retain the potential to differentiate to derivatives of all three embryonic germ layers, whilst maintaining the self-renewal ability *in vitro* (Takahashi et al., 2007; Thomson et al., 1998). Whilst PSCs represent a potent tool in modelling development, they have become a focus of research in their own right, particularly as PSCs provide an unlimited source of differentiated cells for replacement of missing or diseased cells of a patient. The clinical utility of human PSC-derived cellular products necessitates genetic and epigenetic integrity of PSCs and their derivatives, at least to the extent that no mutations that potentially confer malignant properties exist in the transplanted cells (Andrews et al., 2017). Nonetheless, genetic, epigenetic and phenotypic heterogeneity in human PSC cultures is an inevitability of an expanding embryonic-like cellular population. Consequently, relative cellular fitness in PSC cultures plays a major role in determining the composition of human PSC population (Price et al., 2019). With several PSC-based regenerative medicine applications currently in clinical or preclinical phases of development, a thorough understanding of the dynamics of PSC cellular interactions is essential to enabling efficient and safe clinical translation of PSC-derived cell therapies.

Another powerful way of using human PSCs in studying development and in regenerative medicine is through the creation of interspecies chimeras. The introduction of PSCs into a host blastocyst (the so-called human PSC-blastocyst complementation) serves as a novel platform for *in vivo* modelling of early developmental disorders, providing readouts of disease onset and progression, with relevant clinical value (Wu et al., 2016). Moreover, through genetic manipulation, a developmental organ niche could be reserved exclusively for human cells, thereby generating organ-enriched human-animal chimeras, which bodes hope for solving the severe shortage of organ donors worldwide. Whether utilised for embryology research or purposes of regenerative medicine, successful human PSC-blastocyst complementation requires robust human chimerism in host animal species, which has not been achieved due to alleged xenogeneic barriers in early development. Cell competition has also been proposed to act as one of the barriers to interspecies chimerism. During interspecies chimera formation, xenogenic human PSCs may be treated as unfit or aberrant cells and targeted for elimination. PSC competition has not been examined in an interspecies context until most recently.

Here, we discuss the role of cell competition in human PSC populations within several different contexts. First, we speculate on the existence of cell fitness selection during early human embryogenesis, mainly inferred from descriptive studies of human embryos and mechanistic studies from other species. We then discuss the role of cell competition in establishing and expanding PSC cultures, relevant to applications of PSCs in basic biology research and regenerative medicine. Finally, we delve into the recent observations of cell competition in PSCs mixed from different species and consider the implications of understanding cell competition for applications from assisted reproduction to regenerative medicine.

## 2 Cell selection in pluripotent cells of human embryos

The need to understand the exact timings and sequence of early embryonic processes in human development extends beyond academic interest into the area of substantial clinical significance. Indeed, estimates that as many as 70% of human pregnancies do not result in live births speak to the clinical need for unlocking the reasons behind a low fecundity rate in humans (Macklon et al., 2002). The presence of foetal karyotypic abnormalities in the majority of spontaneous miscarriages has prompted the notion that genetic abnormalities are the key contributor to pregnancy loss (Boue et al., 1975; Menasha et al., 2005; Nikitina et al., 2016; Philipp et al., 2003). While errors in meiosis during oogenesis or, less commonly spermatogenesis, account for the whole-embryo aneuploidy (Nagaoka et al., 2012), the advent of preimplantation genetic testing has revealed presence of a widespread mosaic aneuploidy in human embryos (van Echten-Arends et al., 2011; Vanneste et al., 2009). In a recent single-cell RNA-sequencing analysis of human embryos, over 70% of embryos were found to harbour mosaic aneuploidies arising from mitotic errors (Starostik et al., 2020). The susceptibility of early embryonic cells to mitotic errors is counterintuitive with the assumption that embryonic cells have robust mechanisms in place to maintain their genome stability. The observed propensity to aberrant divisions is likely a consequence of the abbreviated cell cycle structure of embryonic cells (Becker et al., 2006). On the one hand, the shortened cell cycle enables rapid proliferation of cells, but on the other hand, it evidently compromises the ability of embryonic cells to control their genetic stability (Ahuja et al., 2016). Similarly, additional exigences of early development may also inadvertently compromise fitness of individual cells in the incipient embryo. After all, early development is synonymous with rapid and profound changes in transcriptome, epigenome, nuclear architecture and metabolism (Shahbazi, 2020). Such a startlingly dynamic nature of early embryogenesis has a clear aftereffect: there is a lot of scope for errors to occur. Thus, it could be argued that the real conundrum in development is not that developmental errors are common, but that normal development can be achieved against the backdrop of high frequency of developmental errors.

Several observations in human embryos may help explain the apparent paradox. First is that the embryos harbouring mosaic aneuploidy can nonetheless result in live births (Fragouli et al., 2017; Greco et al., 2015; Munne et al., 2017) and second, that human preimplantation development is characterized by high incidence of apoptosis (Hardy, 1999). Collectively, these observations suggest an ongoing clearance of genetically aberrant, mis-patterned or otherwise defective cells from developing embryos. In line with this notion, the levels of aneuploidy in human embryos decrease as development progresses (van Echten-Arends et al., 2011). Studies of mosaic mouse embryos have been particularly telling regarding the fates of genetically aberrant cells. Interestingly, in mouse embryos, tetraploid (Sancho et al., 2013) or aneuploid cells (Bolton et al., 2016; Singla et al., 2020) were shown to undergo selective autophagy and apoptosis in the presence of their fitter wild-type counterparts. Such selective attrition of cells with abnormal karyotypes, underpinned by cell competition mechanisms, effectively ensures continuation of normal embryonic development (Sancho et al., 2013; Bolton et al., 2016; Singla et al., 2020).

Apart from the elimination of genetically aberrant cells, cell competition in mouse embryos also selectively ablates cells that in some manner display an inappropriate or less-fit phenotype relative to the rest of their population. The undesired phenotypic features depend on the stage of the embryo development, with different features being selected for in mouse pre-implantation versus post-implantation development. For example, during mouse epiblast differentiation, cell competition eliminates cells that have a relatively low expression of pluripotency factors (Hashimoto and Sasaki, 2019). The expression of pluripotency factors in the mouse epiblast is regulated by TEAD transcription factors, and these in turn are activated by binding to a transcription co-activator YAP. In the mid-blastocyst, YAP expression and, consequently, pluripotency factor expression is heterogeneous within the cells of the ICM. The variegated expression of YAP is resolved by the late blastocyst stage through the process of cell competition, with this quality control mechanism resulting in the retention of cells with high expression of YAP and the concomitantly high expression of pluripotency factors (Hashimoto and Sasaki, 2019).

The quality surveillance for cell fitness also continues into the post-implantation stage of the mouse epiblast. At this stage, cell competition was shown to remove mis-specified cells, such as the ones with the defective Bone Morphogenetic Protein (BMP) signalling, when they are found in a milieu with wild-type cells (Sancho et al., 2013). Moreover, cells with a low expression of Myc are also eliminated from the post-implantation epiblast (Claveria et al., 2013), as are the cells prematurely primed for differentiation (Diaz-Diaz et al., 2017). Different mechanisms have been previously implicated in the sensing and subsequent elimination of loser cells, however, an elegant study has recently revealed the existence of a universal feature underscoring the loser cell phenotypes (Lima et al., 2020). Specifically, phenotypic fingerprints of different types of loser cells, namely the mis-patterned and karyotypically abnormal mouse PSCs, converged on the decreased mitochondrial function. In this study, cell competition could also be induced by disrupting the mitochondrial function, thus pointing to the mitochondrial activity as a key read-out of cellular fitness (Lima et al., 2020).

Based on the data from mouse embryos and other model organisms (reviewed in Baker et al. (2020) and Hashimoto and Sasaki, 2020), it is tempting to speculate that cell competition also plays critical roles in the course of developing a healthy human conceptus. While this remains an open question, several recent advances have opened up the possibility of resolving it empirically. Improvements in the long-term embryo culture have facilitated the study of processes occurring beyond the initial blastocyst formation and during embryo implantation (Deglincerti et al., 2016; Shahbazi et al., 2016; Xiang et al., 2020). Such studies using *in vitro* cultured embryos are being complemented by recently developed synthetic embryos fabricated from PSCs or by assembling multiple cultured stem cell types into embryo-like structures. A stem cell-based human embryo model based on the confinement of PSCs to 2D circular micropatterns mimics some features of post-implantation development, as cells self-organise to generate trophectoderm-like, mesendoderm-like and ectoderm-like layers upon exposure to BMP4 (Warmflash et al., 2014). Another synthetic human embryo model is an epiblast-amniotic sac model, which recapitulates features of gastrulation, the pro-amniotic cavity formation and generation of epiblast-amniotic ectoderm pattern (Shao et al., 2017; Zheng et al., 2019). Finally, a PSC-based gastruloid model entails three-dimensional aggregates recapitulating the formation of the three embryonic germ layers (Moris et al., 2020). More diverse and advanced embryo models have been created using an array of cultured mouse stem cells, with some potentially possessing the ability to develop into an organism. For example, blastocyst-like structures, termed blastoids, contain cells representative of all three lineages found at that stage of mouse development (Li et al., 2019; Rivron et al., 2018; Sozen et al., 2019). When transplanted into surrogate mice, blastoids were able to implant into the uterus and recapitulate some aspects of development, but lacked the proper organisation of extraembryonic and embryonic tissue (Li et al., 2019). Excitingly, two recent studies reported the generation of human blastoids *in vitro*, which resembled human blastocysts in morphology, lineage composition and gene expression profiles, and they developed to peri-implantation embryo-like structures (Yu et al., 2021a; Liu et al., 2021). Notwithstanding the need to optimize the protocols for robust and efficient derivation of human blastoids, these models provide a compelling means to delineating critical steps of early human embryogenesis. Understandably, any technological increments made in this field must operate within a framework of rigorous ethical scrutiny and under well-defined and coherent national and international guidelines (Hyun et al., 2020).

## 3 Dynamic pluripotent stem cell states and their applications

**Fig. 1:**
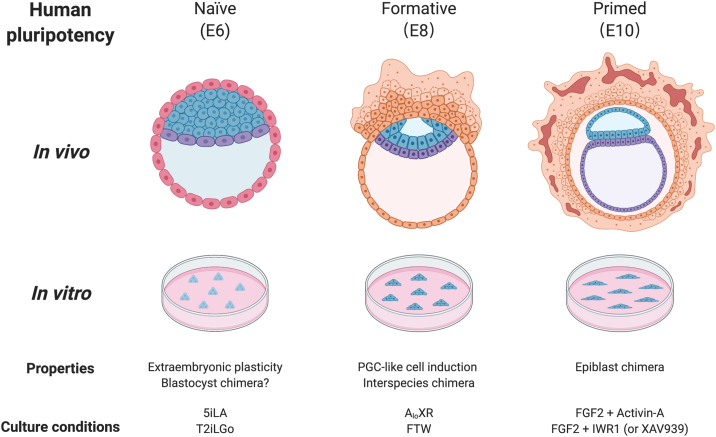
Human pluripotency continuum *in vivo* can be recapitulated in cultured PSCs *in vitro*. By using different conditions naïve, formative and primed human PSCs have been derived from blastocysts and/or somatic cell reprograming, which exhibit distinct functional properties and show transcriptomic similarity to E6, E8 and E10 epiblasts, respectively.

Capturing the embryonic pluripotent cells *in vitro* has been first achieved from mouse blastocyst in 1981 (Evans and Kaufman, 1981; Martin, 1981), but the much-anticipated human equivalents took another 17 years to materialise. When human embryonic stem cells (ESCs) were finally derived in 1998 (Thomson et al., 1998), it became apparent that they show distinct features when compared to their alleged mouse counterparts, even though both were sourced from pre-implantation blastocysts. The first apparent difference is in the colony morphology, which is dome-shaped in mouse and more flattened in human ESCs. The signalling requirements are also distinct, as mouse ESCs rely on leukaemia inhibitory factor (LIF) and BMP signalling (Ying et al., 2003) for self-renewal, whereas human ESCs show dependency on Activin/Nodal and FGF pathways (Vallier et al., 2005) instead. Finally, while mouse ESCs are commonly passaged as single cells, human ESCs show poor clonal survival (Watanabe et al., 2007; Barbaric et al., 2014) and are conventionally passaged as small clumps. These discrepancies were initially attributed to species differences, but were later resolved by the concept of “naïve” and “primed” pluripotent states (Nichols and Smith, 2009). In 2007, two independent groups isolated another PSC type, epiblast stem cells (EpiSCs), from post-implantation mouse epiblasts (Brons et al., 2007, Tesar et al., 2007). Mouse EpiSCs resemble human ESCs in many respects, as they are developmentally more advanced than mouse ESCs and “primed” for lineage differentiation. In addition to molecular and cellular differences, naïve mouse ESCs and primed mouse EpiSCs can also be distinguished by their ability to colonize blastocyst ICMs and contribute to chimera formation (Wu and Izpisua Belmonte, 2015). Mouse ESCs can robustly contribute to chimeras with germline transmission while mouse EpiSCs are limited in this respect. In contrast, mouse EpiSCs (and human ESCs) could engraft to peri-gastrulation epiblast and contribute to chimera formation *ex vivo* (Huang et al., 2012; Mascetti and Pedersen, 2016; Wu et al., 2015) (Fig. 1).

The realization that human ESCs are in the primed pluripotency and the advantages of naïve mouse ESCs over EpiSCs, including higher developmental potential, chimera competency and higher single cell cloning efficiency, have led to the recent development of culture conditions for stabilizing human naïve pluripotency *in vitro*. Due to a lack of gold standard functional test of naïve pluripotency in humans, a flurry of papers reported the generation of human PSCs showing some degree of resemblance to mouse ESCs (Weinberger et al., 2016, Wu et al., 2015). To date, two such human naïve conditions demonstrate most promising results based on molecular criteria (Takashima et al., 2014, Theunissen et al., 2016) and plasticity towards trophoblast cells, a putative feature of epiblast in human blastocysts (Cinkornpumin et al., 2020, Dong et al., 2020, Guo et al., 2021) (Fig. 1). Despite these advances, however, naïve human PSCs generated with current methods seem to display epigenetic instability, which has impaired their widespread adoption in biomedical research (Pastor et al., 2016). Future studies are warrantied to further optimize human naïve PSC cultures to faithfully recapitulate the epigenome of *in vivo* human epiblast cells.

A number of recent studies have reported that human and mouse PSCs putatively reside in intermediate states between naïve and primed pluripotency (Bao et al., 2018, Du et al., 2018, Kinoshita et al., 2021, Neagu et al., 2020, Tsukiyama and Ohinata, 2014, Yu et al., 2021b, Cornacchia et al., 2019; Wu et al., 2015). Several concepts have emerged to define these intermediate states among which formative pluripotency has garnered most attention. Formative pluripotency represents an important time window during early development when naïve pluripotency is reconfigured to prepare for multilineage competency, including germ cells (Smith, 2017). Functionally, formative pluripotency is characterized by both chimera competency and primordial germ cell (PGCs) responsiveness, which is unique to mouse E5-6 epiblasts (Fig. 1). Most recently, two studies reported the generation of stable PSCs with features of formative pluripotency (Kinoshita et al., 2021, Yu et al., 2021b). In one study, by stimulating the FGF and TGF-β pathways and strongly activating canonical WNT signalling (FTW culture), Yu et al. derived intermediate ESCs (from herein referred to as FTW-ESCs) from mouse blastocysts that share transcriptomic similarities with E5-6 epiblast and retain high competence for direct PGC-like cell induction *in vitro* and germline chimera formation *in vivo*. The same culture condition also supported the derivation of ESCs from horse blastocysts and generated transgene-free iPSCs from both horse and human fibroblasts. Horse FTW-ESCs/iPSCs transcriptionally resembled their mouse counterpart, could be directly induced into PGC-like cells (PGC-LCs) by BMP treatment, and contributed to chimera formation in mouse embryos. Human FTW-iPSCs also harboured intermediate pluripotency features and demonstrated PGC responsiveness *in vitro*. Interestingly, in another study, Kinoshita et al. (2021) generated mouse and human stem cells with features of formative pluripotency using a different culture condition containing a low concentration of Activin-A, the canonical WNT pathway inhibitor XAV939, and a pan-retinoic acid receptor inverse agonist (RARi, BMS493) (referred to as A_lo_XR-PSCs here). A_lo_XR-PSCs share similar properties with FTW-PSCs, which include competence for germline specification, chimera formation, and transcriptome similarity to E5-6 epiblasts, among others. There are, however, noticeable differences between A_lo_XR-PSCs and FTW-PSCs. For example, female FTW-PSCs retain two active X-chromosomes (XaXa) while one of the X-chromosomes in female A_lo_XR-PSCs is inactive (XaXi). As random X-chromosome inactivation (XCI) is not fully established by E6.5 (Shiura and Abe, 2019), A_lo_XR-PSCs and FTW-PSCs may represent different types of formative cells between E5-6, or FTW-PSCs and A_lo_XR-PSCs may resemble an early and late formative states, respectively. Overall, these findings suggest that formative pluripotency is a continuum rather than a singular state and demonstrate that different types of PSCs with formative features can be recapitulated *in vitro* under different culture conditions.

Naïve, formative and primed human PSCs represent *in vitro* adaptations of human pluripotency continuum *in vivo* and provide us with readily accessible, inexhaustible, scalable and manipulatable resources for modelling human development and diseases, and for generating cells and tissues for regenerative medicine. Choice of which PSC type to use will likely depend on the application. For example, formative human PSCs are ideal for studying molecular mechanisms underlying human germ cell development and infertility. Naïve human PSCs are supposedly chimera competent, and therefore were believed to be the cells to use for interspecies blastocyst complementation for generating human tissues in animals. However, low chimeric contribution of naïve human PSCs in animals, even at the embryonic stage, has been reported (Theunissen et al., 2016, Wu et al., 2017), which may be attributable to suboptimal naïve human culture condition and/or interspecies developmental incompatibility and xenogeneic barriers (see below). Recently, a newfound feature, plasticity toward extraembryonic lineages, has breathed new life into naïve human PSC research and opened the door for studying earliest human lineage specification and differentiation, and modelling human pre/peri-implantation development *in vitro* (Cinkornpumin et al., 2020, Dong et al., 2020, Guo et al., 2021, Linneberg-Agerholm et al., 2019). Finally, largely due to their long history, primed PSCs have become the first choice for most, if not all, *in vitro* differentiation protocols. A number of primed human PSCs-derived cellular products are currently undergoing clinical trials (Trounson and DeWitt, 2016).

## 4 Cell competition in primed human pluripotent stem cell cultures

While the exceptional abilities of human PSCs to endlessly proliferate and extensively differentiate *in vitro* are recognised as key advantages of using these cells in basic and translational research, the observation that PSCs may harbour genetic changes altering their behaviour warrants caution (Andrews et al., 2017; Halliwell et al., 2020a). In some cases, genetic aberrations can be traced back to the cell-of-origin from which PSCs were originally derived, i.e. ICM and somatic cells for ESCs and iPSCs, respectively. All of the progeny derived from a variant cell-of-origin contains that particular genetic variant, making it easier to detect it in subsequent cultures. In other instances, however, genetic changes may appear after the initial derivation, and accumulate following extended culture period (Baker et al., 2016; Draper et al., 2004). These so-called culture-acquired genetic changes create genetic and phenotypic heterogeneity in PSC populations.

A pertinent question regarding the appearance of culture-acquired genetic changes relates to the frequency of their appearance. Time-lapse tracking of mitoses revealed a relatively high frequency of mitotic errors occurring in PSCs (Halliwell et al., 2020b; Lamm et al., 2016; Zhang et al., 2019). This observation parallels findings of high rates of mosaic aneuploidy in early human embryos (van Echten-Arends et al., 2011; Vanneste et al., 2009; Starostik et al., 2020), suggesting that susceptibility to mitotic errors may not be simply explained as an artefact of an *in vitro* environment, but also reflects an intrinsic property of early embryonic cells. Nonetheless, the observed high frequency of mitotic errors in PSCs does not equate to a particularly unstable genotype. Evidently, PSC lines can be maintained for long periods of time with a stable, euploid karyotype (International Stem Cell et al., 2011). The discordance in the mitotic mutation rate and the frequency of detection of karyotypic changes in PSC cultures suggests an ongoing elimination of aberrant cells from cultures, but whether such aberrations trigger apoptosis intrinsically or whether cell selection mechanisms play a role in this process remains unknown.

**Fig. 2:**
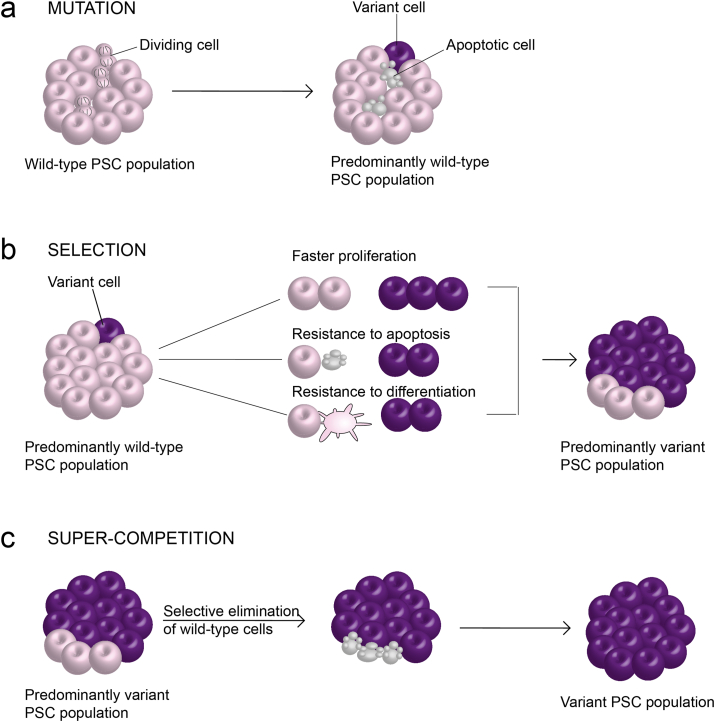
Appearance and selection of genetically variant cells in human PSC cultures. a) Human PSCs are susceptible to mitotic errors, but high levels of apoptosis in PSCs sustain an overall low mutation rate. b) Rare variants that appear in human PSC cultures may overtake the cultures if they exhibit a selective advantage, such as the faster proliferation compared to wilds-type cells, increased resistance to apoptosis or increased resistance to differentiation in comparison to wild-types. c) In some instances, variant cells have the ability to selectively eliminate wild-type cells from mosaic cultures. The result of this process, termed super-competition, is a rapid overtake of culture by the variants.

In theory, the relatively low mutation rate in PSCs provides an excellent grounding for the therapeutic use of PSC-derived cellular product. Yet, both research and clinical applications of PSC inevitably entail expansion of large numbers of cells and keeping them in culture for long periods of time. As a corollary, even a small mutation rate is likely to produce enough genetic heterogeneity in PSC cultures for the next important step in their ‘evolution’ - the selection. While the majority of aberrations seem to be effectively cleared from stem cell populations, thus maintaining an overall low mutation rate (Zhang et al., 2019) (Fig. 2a), occasionally PSCs with the acquired genetic changes become enriched in cultures over time (Draper et al., 2004; International Stem Cell et al., 2011) (Fig. 2b). This happens in instances when the acquired aberration confers human PSCs with a growth advantage, allowing them to outcompete wild-type cells in culture. Systematic analysis of twenty years’ worth of karyotyping data demonstrated a clear non-random trend in the genetic changes that detected in these cells, with gains of regions of chromosomes 1, 17, 20 and gains of whole chromosomes 12 and X being particularly prevalent (reviewed in (Halliwell et al., 2020a)). The same karyotypic abnormalities are a characteristic of embryonal carcinoma cells, the pluripotent stem cell population of germ cell tumours, and are frequently found in many other cancers (Andrews et al., 2005). The apparent link between the genetic changes in PSCs and altered behaviour of variant cells has sparked concerns around the safety of PSC-based cellular therapies. A major worrying aspect lies in the possibility of variant PSCs that skipped detection may compromise the functionality of the final cellular product or render it tumorigenic upon transplantation into a patient.

A prerequisite for preventing the aberrant cells from overtaking the cultures is understanding the mechanisms of their selective advantage. Although a systematic comparison of variant cells is overall lacking, a number of studies have described the impacts of some frequently gained chromosomal regions on the behaviour of variant cells harbouring them. Variant phenotypes are often complex and include to a varying degree the following elements: the resistance to apoptosis, an altered propensity to exit pluripotency and/or for differentiation to particular lineages and a shortened cell cycle (Avery et al., 2013; Barbaric et al., 2014; Ben-David et al., 2014; Fazeli et al., 2011; Werbowetski-Ogilvie et al., 2009). Any of these properties, either alone or in combination, can contribute to the growth advantage of variant cells. Nonetheless, the observed overtake of variants in cultures was sometimes too rapid to be explained solely by cell autonomous effect (Price et al., 2019), and suggests existence of intricate and complex cellular interactions between wild-type and variant cells within the same PSC culture.

We have recently investigated the behaviour of wild-type human PSCs when cultured with variant PSCs harbouring some of the commonly acquired genetic changes (Price et al., 2019). Wild-type cells grown in their homotypic culture grew slower than variants, but nonetheless established viable, proliferative colonies. Strikingly, when cultured in the presence of variants, wild-type cells were selectively eliminated from co-cultures. Time-lapse microscopy revealed corralling of wild-type cells by faster growing variants, causing the wild-types to aggregate within areas of high local cell density. Consequently, the transcription factor coactivator YAP relocated from the nucleus of wild-type cells to their cytoplasm, rendering it inactive. YAP was retained in the nucleus of neighbouring variant cells, which continued to proliferate while wild-type cells underwent apoptosis. This example of variant cell dominance demonstrated the existence of cell competition in cultured human PSCs and raised a possibility that similar selection principles may be at play with other types of genetic changes occurring in PSCs. For example, culture-acquired single nucleotide variants in *TP53* were also noted in PSCs (Merkle et al., 2017), but how *TP53* mutant cells achieve dominance in PSC cultures remains unknown. Moreover, a question arises whether cell competition takes place between wild-type human PSC clones with no overt genetic changes, but with different levels of fitness, as was previously noted in mouse PSCs (Shakiba et al., 2019). Overall, recent findings of the mechanisms of fitness selection in human PSCs highlight the need for a revised model that captures not only intrinsic properties of PSCs, but also cell-cell interactions (Fig. 2c). It is only by recognising and understanding such interactions that we will be in a position to rationally design strategies aimed at expanding large numbers of genetically and phenotypically consistent PSCs for their use in applications from basic biology to regenerative medicine.

## 5 Cell competition as a barrier to interspecies chimerism

Although PSC-based regenerative medicine approaches offer hope for treatment of numerous degenerative diseases (Trounson and DeWitt, 2016), it remains unfeasible to generate entire organs from PSCs *in vitro.* Yet, organ replacement remains an area of an enormous unmet clinical need. As of September 2020, in the United States alone there are over 109,000 men, women, and children on the organ transplant waiting list (https://www.organdonor.gov/statistics-stories/statistics.html). This shortage extends worldwide, and researchers are currently working on a variety of ways to increase the number of organs available. Classical developmental studies conventionally used to assess developmental plasticity of cells have inadvertently paved the way to achieving this feat. Specifically, an *in vivo* approach known as interspecies blastocyst complementation involves the injection of donor PSCs from one species into an organogenesis-disabled blastocyst of a different species. The growing mutant host embryo provides an emptied developmental organ niche exclusively for donor cells to fill (Wu et al., 2016), and thereby generating a PSC-derived organ from the donor species. In addition to regenerative medicine applications, interspecies blastocyst complementation can serve as a useful model system for studying development, e.g., organ and body size control, developmental timing, maternal-fetal communication, and species differences, in an evolutionary context.

The ability of donor PSCs to robustly contribute to chimera formation in the host embryos constitutes a necessary requisite for successful interspecies blastocyst complementation. Rat and mouse PSCs could efficiently differentiate into a number of different cell types inside a growing mouse and rat embryo, respectively, which enabled interspecies organogenesis via blastocyst complementation, e.g., a rat pancreas generated in mice (Kobayashi et al., 2010). Of note is that the host embryos govern many aspects of organ development from the donor species, including size, developmental pace, and even the ability to form an organ non-existent in the host species (e.g., although rats lack a gall bladder, mouse embryo can induce the formation of a gall bladder from the donor rat cells) (Wu et al., 2017). One interesting finding in recent years has been that little to no chimerism could be detected if the donor cells are taken from a species with significant evolutionary divergence from the host. For example, in contrast to chimera formation between different mice and rats, it was difficult for human cells to integrate and develop normally within a mouse or pig embryo (Theunissen et al., 2016; Wu et al., 2017). The majority of donor cells in such cases are eliminated early on in development, suggesting the existence of xenogeneic barriers between evolutionarily distant species. Studying and overcoming these barriers will bring us closer to creating host embryos with donor cells from a distant species that persist until a later developmental stage.

**Fig. 3:**
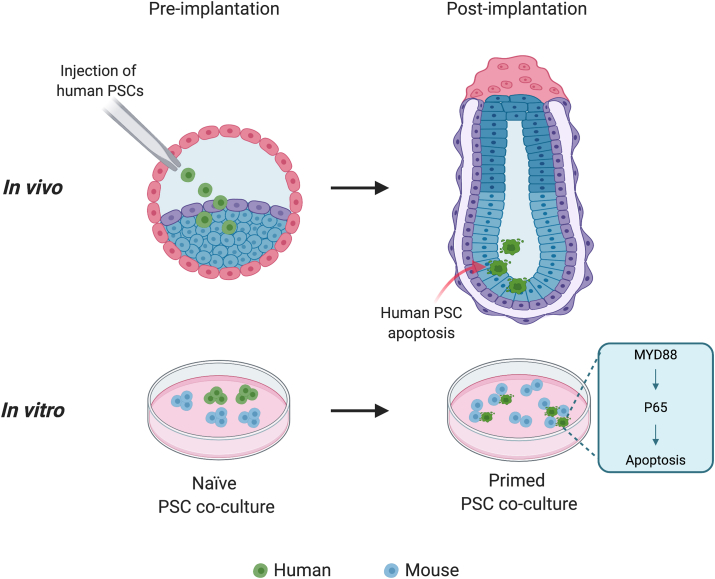
Cell competition has been observed in primed but not naïve interspecies PSC co-cultures between human and mouse, which helps explain the low human cell chimerism in early mouse embryos. Activation of NFκB pathway via MYD88 triggers human cell apoptosis.

Cell apoptosis has been shown by several recent studies to be one of the initial barriers to human chimerism in animals. By blocking donor cell apoptosis, these studies demonstrated improved chimeric contribution of human cells to early embryos from several species including mice, pigs and rabbits (Das et al., 2020, Huang et al., 2018, Wang et al., 2018). It remains unclear, however, whether the elimination of human cells is due to cell-autonomous mechanisms or their competitive interaction with host cells. It is likely that during interspecies chimera formation donor PSCs are treated as unfit or aberrant cells targeted for elimination. To examine interspecies cell competition during early development, we have established an *in vitro* system based on co-culture of PSCs from different species. From these PSC co-culture experiments, it became evident that cell competition occurred between mouse and human cells in a dramatic fashion: most, if not all, human PSCs were actively sought after by mouse PSCs and died within a few days of co-culturing (Zheng et al., 2021). This competitive interaction seems to be specific for primed pluripotency, as co-culturing human and mouse PSCs in naïve and differentiation conditions did not result in overt human cell death (Fig. 3).

To gain insight into the molecular mechanisms underlying human-mouse primed PSC competition, we performed RNA-sequencing of human PSCs either separately cultured or cultured with mouse PSCs. Comparative analysis identified NF-κB signalling pathway was activated in co-cultured human PSCs. Consistent with this finding, knockout of *P65*, a key component of the canonical NF-κB pathway, in human PSCs could overcome human-mouse primed PSC competition. MyD88 is a key adaptor protein for most mammalian Toll-like receptors (TLRs), which has the main role of activating NF-κB. We found that MYD88 deficiency could also rescue human PSCs from being outcompeted by mouse EpiSCs. Suppression of human-mouse primed PSC competition conferred primed human PSCs with the ability to contribute to chimera formation in early mouse embryos.

In addition to human and mouse, we also performed co-culture experiments using primed PSCs from several other species including rat, rhesus macaque and cow. Similar to human-mouse, cell competition could be observed in any co-culture combinations between a primate (human or rhesus) and a rodent (mouse or rat), but not between human and rhesus, or mouse and rat. Interestingly, we found primed bovine ESCs were outcompeted by both rodent and primate PSCs. These results suggest primed PSC competition is a general phenomenon among species with differential cell fitness. Similar to mouse EpiSCs, *MYD88* and *P65* deficiency also prevented human PSCs from being eliminated by rat EpiSCs during co-culture. It remains to be determined if TLR/NFκB pathway plays a conserved role in primed PSC competition between other species.

Through interspecies PSC co-culture experiments, our study has uncovered a previously unrecognized mode of cell competition seemingly between PSCs of evolutionarily distant species during primed pluripotency. This unique interspecies cell competition model may help uncover novel molecular mechanisms generalizable to other cell competition contexts. Moreover, much remains to be understood why the interspecies competition happens during the primed pluripotency phase and how such a mechanism could have evolved. One speculation is that primed PSCs correspond to highly proliferative peri-gastrulation epiblast cells that undergo rewiring of their transcriptional, epigenetic, metabolic and signaling networks in preparation for differentiation to establish distinct cell lineages. To ensure unperturbed development, epiblast cells at this stage may have evolved a mechanism, putatively through innate immune system (TLRs/Myd88) to activate NFκB-dependent apoptosis in “aberrant” or “unfit” cells, and thereby preventing them from further participation in development. TLRs/NFκB is the key signaling pathway mediating the ancient and conserved innate immune recognition system activated in response to altered-self cells (Meyer et al., 2014), which may underlie the response to eliminate less-fit cells during interspecies primed PSCs competition. Of note is that components of the innate immune system (Toll-related receptors, TRRs) have also been shown to eliminate unfit cells through NFκB-dependent apoptosis during cell competition in *Drosophila* wing disc development (Meyer et al., 2014), and thus innate immunity/NFκB pathway may act as a conserved gatekeeper to ensure normal development. By understanding the molecular mechanisms and overcoming interspecies PSC competition donor cell chimerism in a distant host species can be further improved, and thereby holding great potential to realize the dream of generating human organs in animals to solve the world-wide shortage of donor organs.

## 6 Conclusion

Cell competition, a quality control mechanism that scrutinises cellular fitness and removes less-fit cells from heterogeneous populations, has been demonstrated in many different cellular populations *in vitro* and *in vivo*. Therefore, recent findings implicating cell competition in determining the sub-clonal composition of human PSC populations came as no surprise to developmental biologists. Nonetheless, surprising was the diversity of cell competition mechanisms operating in different PSC contexts. The variety of these mechanisms likely reflects the exceptionally dynamic nature of pluripotency, with each pluripotent state favouring selection of specific cellular features. The dynamic states of pluripotency during development, which have also been captured in equivalent naïve, formative and primed states *in vitro*, therefore provide an exceptional model for exploring molecular rules of cell fitness selection. The use of complementation experiments, either through mixing with xenogeneic cells *in vitro* or by introducing PSCs into host embryos of different species, offers an additional route into the understanding of the molecular machinery of PSC cell competition. Ultimately, deciphering how cell fitness is sensed within a population and how elimination of unfit cells is executed, will have a significant impact on our ability to control stem cell fates in applications ranging from basic research and disease modelling through to regenerative medicine. Finally, a recent upsurge in PSC-based models of human conceptus offers an opportunity to delineate how cell selection contributes to upholding the cellular fitness in what is undoubtedly one of the most enigmatic contexts in biology – the human embryo.
